# Associations between excessive supragastric belching and esophageal reflux factors in patients with PPI-refractory GERD in Japan

**DOI:** 10.1007/s00535-025-02258-4

**Published:** 2025-05-12

**Authors:** Yukihiro Shuto, Masahiro Saito, Tomoyuki Koike, Kaoru Koizumi, Yumiko Kaise, Kazuma Yachi, Yutaka Hatayama, Yohei Ogata, Xiaoyi Jin, Takeshi Kanno, Waku Hatta, Kaname Uno, Naoki Asano, Akira Imatani, Atsushi Masamune

**Affiliations:** Division of Gastroenterology, Tohoku University Graduate School of Medicine, 1-1 Seiryo-machi, Aoba-ku, Sendai, Miyagi Japan

**Keywords:** Supragastric belching, Esophageal reflux, PPI-refractory GERD, 24-h multichannel intraluminal impedance-pH monitoring, High-resolution impedance manometry

## Abstract

**Background:**

No studies have evaluated the prevalence of supragastric belching (SGB) in Japanese patients with proton pump inhibitor (PPI)-refractory non-erosive reflux disease (NERD) under off-PPI conditions. This study aimed to clarify the association between excessive SGB and esophageal reflux factors.

**Methods:**

Seventy-nine patients with PPI-refractory NERD under off-PPI treatment were evaluated using 24-h multichannel intraluminal impedance pH monitoring and high-resolution impedance manometry.

**Results:**

The prevalence values of excessive SGB overall and in the true NERD, reflux hypersensitivity, and function heartburn subtypes were 19.0%, 35.7%, 5.3%, and 12.5%, respectively. The monitoring results demonstrated that, compared with those without excessive SGB, patients with excessive SGB had a significantly higher total number of reflux events (63 episodes vs. 39 episodes, *p* = 0.01) and significantly greater acid exposure time (6.1% vs. 1.35%, *p* = 0.01). However, bolus exposure did not differ significantly between the groups (*p* = 0.09). The manometry findings showed no significant differences in lower esophageal sphincter pressure, integrated relaxation pressure, and distal contractile integral between the groups. Regarding gastroesophageal reflux, 22% of the SGB episodes were preceded by reflux, 55% occurred independently, and 23% were followed by reflux.

**Conclusions:**

The prevalence of excessive SGB in Japanese patients with PPI-refractory NERD under off-PPI conditions was 19.0% and most commonly observed in patients with true NERD (35.7%). Patients with excessive SGB exhibited increased esophageal acid exposure, and reflux events were sometimes observed before SGB episodes.

**Supplementary Information:**

The online version contains supplementary material available at 10.1007/s00535-025-02258-4.

## Introduction

Gastroesophageal reflux disease (GERD), characterized by heartburn and regurgitation, is treated using proton pump inhibitors (PPI). However, many patients with PPI-refractory GERD experience symptoms that are not sufficiently controlled [1]. Among these, supragastric belching (SGB) is a pathophysiological condition associated with PPI-refractory GERD, which was recently reported in 42% of these patients [2, 3].

Belching, the expulsion of air from stomach or esophagus, is common but can affect quality of life when it occurs frequently [4, 5]. Based on the findings of multichannel intraluminal impedance-pH (MII-pH) monitoring, belching can be classified into two types: gastric belching and SGB [2]. Gastric belching involves air accumulation in the proximal stomach, increased gastric pressure, and stretching of the gastric wall, which relaxes the lower esophageal sphincter (LES). This allows air to reflux into the esophagus, subsequently opening the upper esophageal sphincter (UES) to release air through the mouth [2]. In contrast, SGB involves the unconscious inhalation of air into the pharynx, simultaneously relaxing the UES and allowing air to enter the esophagus and be expelled shortly after as belching [2].

Previous studies have reported that 9.3–28.8% of the general population experiences belching, with higher rates in patients with GERD [6–10]. These reports included both gastric belching and SGB, and similar to the prevalence of GERD itself, the proportion of patients with GERD and excessive SGB varies regionally [11].

SGB is a potential cause of symptoms in some patients with PPI-refractory GERD [2]. In patients with GERD and SGB, this air entry can trigger gastroesophageal junction relaxation, inducing reflux symptoms such as heartburn [3, 12]. A study at the Royal London Hospital reported that 35% of patients with PPI-refractory GERD had SGB linked to reflux symptoms [3, 13]. Patients with excessive SGB respond poorly to acid-suppressive therapy but may benefit from cognitive behavioral therapy and diaphragmatic breathing training [12]. Therefore, identifying excessive SGB is crucial for determining effective treatment strategies.

Most studies on SGB have originated in Western countries, with few reports from Asia [14, 15]. A recent Japanese study reported that 18.5% of patients with PPI-refractory GERD had excessive SGB following PPI or potassium-competitive acid blocker (P-CAB) treatment (on-PPI) [16]. However, no studies have reported on the prevalence of excessive SGB, esophageal motility, and reflux under off-PPI conditions in Japanese patients. Clarifying these factors will lead to more effective treatment strategies for PPI-refractory GERD and excessive SGB.

This study aimed to determine (1) prevalence of excessive SGB in Japanese patients with PPI-refractory non-erosive reflux disease (NERD) under off-PPI treatment, (2) characteristic symptoms of excessive SGB, (3) esophageal motility and reflux factors associated with excessive SGB, and (4) relationship between SGB and reflux.

## Methods

### Subjects

**Fig. 1:**
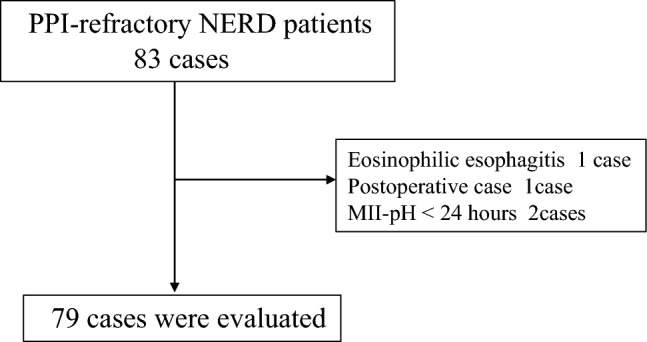
Flowchart of the study population. Among 83 patients with PPI-refractory NERD initially enrolled, four cases were excluded: one patient was diagnosed with eosinophilic esophagitis, one was a postoperative case, and two discontinued MII-pH monitoring before completing 24 h. Consequently, the final analysis included 79 patients

This study retrospectively analyzed 83 patients with PPI-refractory NERD who visited Tohoku University Hospital between July 2019 and July 2024. These patients presented with typical reflux symptoms, such as heartburn, regurgitation, and chest pain. All patients underwent upper endoscopy either prior to the initiation of PPI or P-CAB therapy, or after discontinuation of these medications for at least two weeks. They underwent 24-h MII-pH monitoring and high-resolution impedance manometry (HRIM) under off-PPI conditions, within six months of endoscopic examination. After excluding four cases (one patient with eosinophilic esophagitis, one postoperative case, and two who discontinued MII-pH monitoring before 24 h), the final study population included 79 patients (Fig. 1).

This study was approved by the Ethics Committee of the Tohoku University Graduate School of Medicine (2024-1-421).

### Patients with PPI-refractory NERD

Patients with PPI-refractory NERD were defined as those who continued to experience heartburn, regurgitation, or chest pain despite at least 8 weeks of optimized acid-suppressive therapy, consisting of either a double-dose PPI regimen (rabeprazole 20 mg/day) or a standard dose of a P-CAB (vonoprazan 20 mg/day). Specifically, patients with a Frequency Scale for the Symptoms of GERD (FSSG) score of 8 or higher following treatment were classified as PPI-refractory [17, 18].

### 24-h MII-pH monitoring

Patients underwent 24-h MII-pH monitoring under off-PPI conditions (without PPI or P-CAB treatment for at least two weeks) using a ComforTec MII-pH catheter (ZAN-BG-44, Sandhill Scientific Inc., Highland Ranch, CO, USA).

Before MII-pH monitoring, an HRIM catheter was inserted transnasally to locate the LES. The MII-pH catheter included two pH sensors for measuring esophageal and gastric pH, as well as six impedance sensors. The pH electrodes were calibrated with pH 4.0 and pH 7.0 buffer solutions (Sandhill Scientific Inc.) before transnasal insertion. The catheter was positioned with the esophageal pH sensor placed 5 cm above the LES and the impedance channels at 3, 5, 7, 9, 15, and 17 cm above the LES. The placement of the pH sensor was confirmed via chest radiography. Data were recorded using a portable recording device, and patients were instructed to maintain their usual diet and daily activities during the monitoring period. The patients were also asked to press the event button on the device if reflux symptoms occurred during the recording period. The catheter was removed after 24 h of monitoring [19].

### Analysis of 24-h MII-pH monitoring data

All MII-pH recordings were analyzed using BioVIEW^®^ software (Sandhill Scientific Inc.) with Auto Scan, followed by manual review [20]. The presence of liquid or gas in the esophagus was recorded as decreased and increased impedance, respectively. Liquid reflux was defined as a reduction in impedance by ≥ 50% from baseline, propagating in a retrograde manner across at least two consecutive sensors from the most distal impedance sensor. Acid reflux was defined as reflux with a drop in pH to < 4, or if the pre-reflux pH was already < 4, maintaining a pH < 4 during the event. Non-acid reflux was defined as liquid reflux with pH ≥ 4. Acid exposure time (AET) was calculated as the percentage of time with pH < 4 during the 24-h recording, with abnormal AET set at > 4.2% under off-PPI conditions, based on previous studies [20]. Liquid reflux, as measured for bolus exposure, was defined as a decrease in impedance of > 50% from baseline at the most distal impedance sensor (3 cm above the LES) and propagating retrogradely through at least two consecutive sensors [21].

The patients were instructed to remain upright during the day, either sitting or standing, until bedtime. The time spent upright was defined as “daytime,” while the time spent in the supine position during sleep was defined as “nighttime.”

### SGB in MII-pH

Bredenoord et al. defined SGB during pH-impedance monitoring as a rapid increase in impedance of > 1000 ohms above baseline, followed by a rapid return to baseline in a retrograde manner [2]. This indicates that air swallowed into the distal esophagus quickly returns to the proximal esophagus, distinguishing it from gastric belching, in which air moves from the stomach to the proximal esophagus and is expelled through the mouth [22]. In the present study, we manually counted the number of SGB events over 24 h to ensure that gastric belching was not included in SGB.

### Excessive SGB and SGB severity

Based on previous studies, patients with > 13 SGB events during 24-h MII-pH monitoring were classified as having excessive SGB [16, 23]. The severity of excessive SGB over 24 h was also categorized as mild (14–49 episodes), moderate (50–99 episodes), or severe (≥ 100 episodes) [24].

### Reflux in relation to SGB

SGB occurring within 5 s after a reflux event was defined as “SGB during reflux,” while SGB not associated with reflux was termed “SGB without reflux.” SGB followed by reflux within 5 s was defined as “SGB-inducing reflux” [24].

SGB-induced reflux and its contribution to AET were also assessed as described above. We evaluated the contribution of SGB-induced reflux to the total AET. The acid exposure associated with these events was measured and the proportion of AET attributable to SGB-induced reflux was calculated as a percentage of the total AET in each patient. The proportion of AET related to SGB-induced reflux was then analyzed in the excessive SGB group.

### NERD subtypes

Based on the MII-pH monitoring results under off-PPI conditions, patients were classified according to esophageal AET findings and the association between reflux events and symptoms, as previously described.

We assessed reflux symptom association using the Symptom Index (SI) and Symptom Association Probability (SAP) for typical esophageal reflux symptoms. SI is defined as the proportion of reflux-related symptoms relative to the total number of symptoms. SAP, calculated using Fisher’s exact test, determines the probability of symptom–reflux association by evaluating whether each consecutive 2-min period includes a symptom and/or reflux. A positive symptom–reflux association was defined as either SI > 50% or SAP > 95% [25, 26].

We classified the patients into NERD subtypes based on AET and the presence of symptom association indices. True NERD was defined as an AET of ≥ 4.2%. Patients with an AET of < 4.2% but with a positive SI or SAP were classified as having reflux hypersensitivity (RH), whereas patients with an AET of < 4.2% and negative SI and SAP were categorized as having functional heartburn (FH) [27].

### Frequency Scale for the Symptoms of GERD (FSSG) Questionnaire

On the same day as MII-pH monitoring and HRIM, patients completed the self-administered FSSG to assess their symptoms. Each symptom was scored based on its frequency: none (0), rarely (1), sometimes (2), often (3), or always (4) [18]. The reflux-related score was calculated as the sum of seven specific items (Nos. 1, 4, 6, 7, 9, 10, and 12), while the dyspepsia-related score was based on five items (Nos. 2, 3, 5, 8, and 11). The total score represented the sum of all 12 questionnaire items.

### High-resolution manometry

HRM was performed using a catheter and measurement system (InSIGHT G3^®^, Sandhill Scientific Inc.) equipped with 32 intraluminal pressure sensors and eight impedance channels (four pairs). The test was conducted according to the Chicago Classification version 4.0 protocol. Resting LES pressure was measured by inserting a catheter with the patient in a seated position, without swallowing for 20 s. The baseline LES pressure was recorded at the end expiration [28]. The integrated relaxation pressure (IRP) is a measure of deglutitive relaxation, defined as the lowest mean axial pressure over 4 s, whether continuous or discontinuous, across the LES during the 10-s period following a swallow. The distal contractile integral (DCI) was measured as the average value of > 10 swallows with the patient in the supine or right lateral position and > 5 swallows in the sitting position. The DCI values were automatically calculated using software [28]. Ineffective esophageal motility (IEM) was diagnosed according to the criteria from the Chicago Classification version 4.0.

### Evaluation items

A comparative analysis was conducted on the study subjects based on the following parameters. In addition to analyzing all cases, these evaluations were specifically performed for patients with true NERD.

1. Prevalence of excessive SGB, clinical characteristics, and association with NERD subtypes

We calculated the prevalence of excessive SGB among patients with PPI-refractory NERD and analyzed the clinical background of those affected. Additionally, we assessed the severity distribution of excessive SGB based on predefined criteria. Furthermore, we evaluated the prevalence of excessive SGB within different NERD subtypes, including true NERD, RH, and FH.

2. FSSG score analysis

We compared FSSG scores, focusing on the reflux and dyspepsia-related subscales and the total FSSG score, to evaluate the differences in symptoms between patients with and without excessive SGB.

3. Esophageal reflux factors and motility in excessive SGB

We compared factors related to esophageal reflux, as measured by MII-pH monitoring, between patients with and without excessive SGB. We also examined the differences in esophageal motility parameters, as assessed by HRIM, between these two groups.

4. Association between SGB and esophageal reflux

We determined the mean proportion of each SGB type—those preceding reflux, those occurring without reflux, and those inducing reflux—in each patient with excessive SGB.

### Statistical analysis

Continuous and categorical variables are expressed as means ± standard deviation or medians (IQR) and frequency (%), respectively. Categorical variables were compared between groups using the chi-squared test, whereas Fisher’s exact test was used for small sample sizes. For continuous variables that did not follow a normal distribution, the Mann–Whitney *U* test was applied.

All analyses were performed using R software, version 4.4.1 (R Core Team, Vienna, Austria). Statistical significance was set at *p* < 0.05.

## Results

### Clinical characteristics of patients with PPI-refractory NERD and excessive SGB

In 10 out of 79 cases (12.7%) where endoscopic findings prior to the initiation of PPIs or P-CABs could not be confirmed at the initial visit, endoscopic examination was performed after discontinuation of PPI or P-CAB therapy for more than two weeks (median withdrawal period: 5.5 weeks; interquartile range [IQR]: 2–8 weeks).

Excessive SGB was observed in 15 of 79 patients (19.0%). Mean age and BMI did not differ significantly between the excessive and non-excessive SGB groups. However, the excessive SGB group had a significantly higher proportion of men (*p* = 0.006) (Table 1). Among patients with true NERD, the excessive SGB group had a higher proportion of male patients and a younger age. No other background characteristics differed significantly between the two groups (Supplementary Table 1).

**Table 1:** Patient clinical characteristics

	Total	Excessive SGB	Non-excessive SGB	*p* value
Number, *n*	79	15	64	
Male, *n* (%)	33	11 (73.3)	22 (34.4)	0.006
Mean age (SD) (years)	55.2 (16.3)	56.0 (15.1)	55.0 (16.7)	0.85
Mean BMI (SD) (kg/m^2^)	22.8 (4.0)	24.3 (4.1)	22.5 (3.9)	0.12
History of alcohol consumption, *n* (%)	25	3 (20.0)	22 (34.4)	0.28
History of smoking, *n* (%)	10	2 (20.0)	7 (10.9)	0.34
*H. pylori* infection *n* (%)	5	0 (0)	5 (7.8)	0.26

BMI, body mass index; SGB, supragastric belching

### Excessive SGB severity

Mild, moderate, and severe SGB comprised 66.7%, 13.3%, and 20.0% of cases, respectively.

### Distribution of NERD subtypes and prevalence of excessive SGB by NERD subtype

**Fig. 2:**
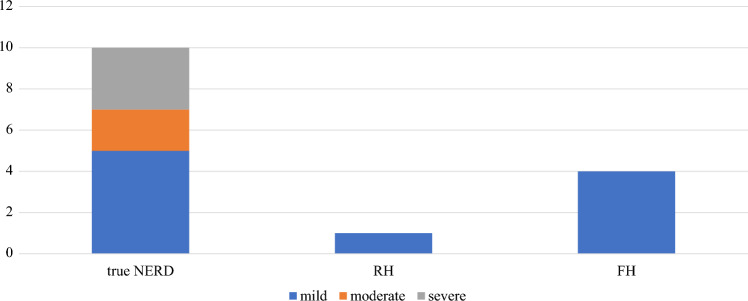
SGB distribution across NERD subtypes and the severity of excessive SGB within each subtype. Of the 15 patients with excessive SGB, 10 (66.6%) had true NERD, 1 (6.7%) had RH, and 4 (26.7%) had FH. The severity over 24 h was categorized as mild (14–49 episodes), moderate (50–99 episodes), or severe (≥ 100 episodes). Moderate to severe SGB was observed predominantly in patients with true NERD

Among the 79 patients with PPI-refractory NERD, 28 (35.4%), 19 (24.1%), and 32 (40.5%) had true NERD, RH, and FH, respectively. The distribution of NERD subtypes in the 15 patients with excessive SGB is illustrated in Fig. 2. Cases with true NERD showed moderate to severe SGB, whereas all cases with RH and FH subtypes showed mild SGB. The prevalence of excessive SGB within each subtype was 35.7%, 5.3%, and 12.5% for true NERD, RH, and FH, respectively.

### Symptom characteristics in the FSSG

There were no significant differences in the total FSSG score between the excessive and non-excessive SGB groups (Table 2). Similarly, the reflux- and dyspepsia-related subscale scores did not differ significantly between the two groups.

**Table 2:** Associations Between F Scale Questionnaire (FSSG) scores and excessive SGB

FSSG score	Excessive SGB	Non-excessive SGB	*p* value
Reflux-related score, median (IQR)	7 (6–13)	11 (7.75–16.5)	0.19
Dyspepsia-related score, median (IQR)	8 (6–9.5)	8 (6–11.25)	0.78
Total score, median (IQR)	15 (13–21)	19.5 (15–28)	0.25

FSSG; Frequency Scale for the Symptoms of GERD, GERD, gastroesophageal reflux disease; SGB, supragastric belching; IQR, interquartile range (25th–75th percentile)

In the analysis limited to patients with true NERD, the total FSSG score did not show a significant difference between the excessive and non-excessive SGB groups. (Supplementary Table 2).

### Characteristics of esophageal reflux factors in MII-pH monitoring

The 24-h AET was significantly higher in the excessive SGB group than in the non-excessive SGB group (*p* = 0.01) (Table 3). Daytime AET was also significantly higher in the excessive SGB group (*p* = 0.02). However, nighttime AET did not differ significantly between the groups (*p* = 0.12). Bolus exposure also did not differ significantly between the excessive and non-excessive SGB groups (*p* = 0.09). The total number of reflux episodes was higher in the excessive SGB group than in the non-excessive SGB group (*p* = 0.01) (Table 3).

**Table 3:** Esophageal acid and bolus exposure times and esophageal reflux episodes

	Excessive SGB	Non-excessive SGB	*p* value
Number*, n*	15	64	
Median (IQR) SGB numbers*, n* (24 h)	26 (17.5–70)	0 (0–1)	
Median (IQR) acid exposure time (%, daytime)	8.5 (2.75–13)	2.1 (0.65–6.35)	0.02
Median (IQR) acid exposure time (%, nighttime)	0.7 (0–8.3)	0 (0–0.6)	0.12
Median (IQR) acid exposure time (%, 24 h)	6.1 (2.45–12.05)	1.35 (0.55–4.525)	0.01
Median (IQR) bolus exposure (%, daytime)	3.9 (1.75–7.15)	2.2 (1.075–3.5)	0.06
Median (IQR) bolus exposure (%, nighttime)	0.2 (0–0.5)	0.1 (0–0.525)	0.69
Median (IQR) bolus exposure (%, 24 h)	1.9 (1.35–4.4)	1.5 (0.7–2.8)	0.09
Median (IQR) acid reflux*, n* (daytime)	36 (27–52.5)	13 (4–29.5)	0.002
Median (IQR) non-acid reflux*, n* (daytime)	22 (11.5–39.5)	17.5 (11–28.75)	0.59
Median (IQR) total reflux*, n* (daytime)	52 (45.5–92.5)	35.5 (22.75–56)	0.01
Median (IQR) acid reflux*, n* (nighttime)	4 (0–6)	1 (0–3)	0.12
Median (IQR) non-acid reflux*, n* (nighttime)	1 (0.5–3.5)	2 (0–3.25)	0.86
Median (IQR) total reflux*, n* (nighttime)	6 (1–10)	3 (1–8)	0.63
Median (IQR) total reflux*, n* (24 h)	63 (50–106.5)	39 (23–63.75)	0.01

AET, acid exposure time; SGB, supragastric belching; IQR, interquartile range (25 th–75 th percentile)

When the analysis was limited to patients with true NERD, AET did not differ significantly between the groups. However, the total reflux episodes and the daytime reflux episodes were higher in the excessive SGB group compared with the non-excessive SGB group (Supplementary Table 3).

### Characteristics of esophageal motility in HRIM

HRIM analysis showed no significant differences in LESP, IRP, or DCI between the excessive and non-excessive SGB groups (Table 4).

**Table 4:** Association between excessive SGB and esophageal motility

	Excessive SGB	Non-excessive SGB	*p* value
Number*, n*	15	64	
Median (IQR) LESP (mmHg)	21.5 (16.2–27.35)	19.7 (10.95–33.125)	0.81
Median (IQR) IRP (mmHg)	8 (5–20)	12.5 (9–21.25)	0.08
Median (IQR) DCI (mmHg-sec-cm)	744 (346–1000)	776 (386.75–1365.25)	0.75
IEM*, n*	4	15	
Non-IEM*, n*	11	49	
Proportion of IEM (%)	26.7	23.4	0.79

DCI, distal contractile integral; IRP, integrated relaxation pressure; LESP, lower esophageal sphincter pressure; IEM, ineffective esophageal motility; SGB, supragastric belching; IQR, interquartile range (25 th–75 th percentile)

Regarding the relationship between excessive SGB and IEM, the prevalence of IEM did not differ significantly between the excessive and non-excessive SGB groups (Table 4). When limited to patients with true NERD, the HRIM results did not differ significantly between the two groups (Supplementary Table 4).

### Reflux in relation to SGB

The distribution of reflux in relation to SGB was as follows: SGB during reflux, without reflux, and SGB-induced reflux accounted for 22.0%, 55.0%, and 23.0% of cases, respectively. In the excessive SGB group, SGB was responsible for 5.5% of the total AET (range 0–22.5% of the 24-h acid exposure).

## Discussion

Studies in Western countries have demonstrated the involvement of SGB in the pathophysiology of PPI-refractory GERD [3, 13]. In Japan, while a previous study reported that 18.5% of patients with PPI-refractory GERD had excessive SGB, this analysis considered on-PPI only [22]. Therefore, the present study aimed to determine the prevalence of excessive SGB in Japanese PPI-refractory NERD patients under off-PPI conditions; to evaluate the impact of SGB on symptoms, esophageal acid exposure, and reflux episodes; and assess the association of SGB with impaired esophageal motility. These findings indicated a 19.0% prevalence of excessive SGB in Japanese patients with PPI-refractory NERD under off-PPI conditions.

Unlike previous studies, the present study was conducted under off-PPI conditions, which allowed for a more accurate assessment of esophageal reflux factors and their association with SGB. On-PPI conditions suppress gastric acid secretion, making it difficult to evaluate reflux episodes, including those preceding or following SGB. Additionally, the off-PPI conditions in the present study enabled a more precise classification of NERD subtypes, facilitating a clearer analysis of the interplay between SGB and reflux.

A key strength of this study was its assessment of the pathophysiology of SGB-related esophageal reflux factors using MII-pH monitoring under off-PPI conditions. The results demonstrated that patients with excessive SGB had higher esophageal AET and a greater number of reflux episodes than those without excessive SGB. The increased esophageal reflux in cases with SGB suggests that even in Japan, SGB is associated with GERD and may serve as an important trigger. Another key finding was that elevated acid reflux episodes and AET were observed only during the daytime, whereas night-time acid reflux and AET were normal. As SGB generally occurs only during the daytime [29], these results support the notion that SGB induces esophageal reflux and that esophageal reflux may, in turn, trigger SGB.

Previous studies conducted overseas have reported IEM in patients with excessive SGB [23]. However, in the present study, we observed no significant differences in test data or IEM prevalence between patients with and without excessive SGB. Sergeev et al. reported an increasing trend in the prevalence of IEM with increasing excessive SGB severity, from 23.4% in patients with mild SGB to 24.9% and 29.6% in those with moderate and severe SGB, respectively [23]. In contrast, in the present study, mild SGB accounted for 66.7% of cases, whereas in Western studies, severe SGB accounted for 51.7% of cases [23]. Thus, the lack of IEM in Japanese patients with excessive SGB may be attributed to the higher proportion of mild cases.

In patients with PPI-refractory NERD, MII-pH monitoring has been used to classify NERD into subtypes according to the Lyon Consensus and Rome Criteria, allowing for a detailed assessment of its pathophysiology [26, 30]. In a previous Japanese on-PPI study [16], the prevalence of excessive SGB in each NERD subtype was as follows: true NERD, 16%; RH, 30%; and FH, 44%. In the present study, the prevalence of excessive SGB within each NERD subtype was 35.7%, 5.3%, and 12.5% for true NERD, RH, and FH, respectively, indicating a substantial proportion of excessive SGB in true NERD cases. The results of the present study demonstrated the highest prevalence of excessive SGB in true NERD, contrary to a previous on-PPI study where it was most common in RH. This discrepancy may be due to differences in NERD subtype classification between on-PPI and off-PPI conditions. Additionally, the results of the present study showed that some SGB episodes were triggered by reflux. Under off-PPI conditions, the increased reflux in true NERD may have induced more SGB, explaining its higher prevalence in this group.

Furthermore, the ability to compare only the true NERD subtype within the NERD classification was also valuable. While the overall comparison between the SGB and non-SGB groups showed a higher incidence of esophageal reflux in the SGB group, the proportion of true NERD, a subtype with a high reflux burden, should also be considered. Comparison of the SGB and non-SGB groups within the true NERD subtype alone showed high levels of reflux in both groups, resulting in no significant difference in AET. However, the total and daytime reflux episodes were higher in the SGB group than in the non-SGB group. The increased number of reflux episodes, particularly during the daytime, was considered to reflect the characteristic of SGB occurring only during the daytime [29]. On the other hand, no significant difference was observed in AET, which may be attributed to the relatively low proportion of AET associated with SGB (5.5%). Therefore, from the perspective of reflux episodes, SGB may be a characteristic factor influencing true NERD.

Regarding reflux in relation to SGB, previous studies focused on on-PPI conditions have shown that isolated SGB (SGB without reflux) is the most common type. However, in this study, reflux preceding SGB (SGB during reflux) accounted for 22% of cases, whereas SGB that triggered reflux (SGB-inducing reflux) accounted for 23% of cases. These findings indicate that under physiological and off-PPI conditions, reflux can trigger SGB, and that SGB itself can also induce reflux. In the excessive SGB group, SGB-induced reflux accounted for 5.5% of the total AET. Although this proportion was lower than that reported by Glasinovic et al., this difference may be attributed to the lower prevalence of severe SGB cases in our cohort compared with the UK population. Furthermore, in cases where SGB did not trigger reflux, the contribution of SGB-related AET was naturally lower [12]. The low proportion of SGB-related AET may be explained not only by the predominance of mild SGB cases in Japan, but also by the possibility that SGB was triggered by discomfort caused by NERD. In fact, 22% of SGB episodes occurred following reflux events, suggesting that reflux-induced discomfort may have contributed to the development of SGB.

This study has several limitations. First, this was a retrospective analysis of MII-pH monitoring, HRIM, and FSSG data from patients attending a single tertiary medical center, which may not be representative of the broader NERD population. Second, the study was conducted exclusively in Japanese patients, and the findings may differ from those observed in Western populations.

In this study, we determined the prevalence of excessive SGB in patients with PPI-refractory NERD and demonstrated the relationship between symptoms and reflux, which could significantly contribute to the management of PPI-refractory NERD. Specifically, as SGB can induce reflux, controlling its occurrence through cognitive behavioral therapy—a treatment for SGB—may help reduce reflux. Therefore, the accurate diagnosis of excessive SGB using MII-pH and the implementation of appropriate treatment could play crucial roles in managing these patients.

In conclusion, the prevalence of excessive SGB in Japanese patients with PPI-refractory NERD undergoing off-PPI treatment was 19.0%, which is similar to previously reported on-PPI findings. Among NERD subtypes, 35.7% of patients with true NERD exhibited excessive SGB. Additionally, patients with excessive SGB exhibited increased esophageal acid reflux, which often preceded SGB occurrence.

## Supplementary Information

Below is the link to the electronic supplementary material.

Supplementary file1 (DOCX 29 KB)

## Abbreviations

AET: Acid exposure time
DCI: Distal contractile integral
FH: Functional heartburn
FSSG: Frequency scale for the symptoms of GERD
GERD: Gastroesophageal reflux disease
HRIM: High-resolution impedance manometry
IRP: Integrated relaxation pressure
LES: Lower esophageal sphincter
LESP: Lower esophageal sphincter pressure
MII-pH monitoring: 24 h multichannel intraluminal impedance pH monitoring
NERD: Non-erosive reflux disease
P-CAB: Potassium-competitive acid blockers
PPI: Proton pump inhibitor
RH: Reflux hypersensitivity
SGB: Supragastric belching
