# Supplementary material for: Associations between excessive supragastric belching and esophageal reflux factors in patients with PPI-refractory GERD in Japan
Source: J Gastroenterol. 2025 May 12;60(9):1070–8. doi: 10.1007/s00535-025-02258-4 (PMC12378752; doi:10.1007/s00535-025-02258-4)
Supplement: Supplementary file 1 — Supplementary file1 (DOCX 29 KB) [file 535_2025_2258_MOESM1_ESM.docx]

Supplementary Table 1. Clinical Characteristics of Patients with True NERD

|  | Excessive SGB | Non-excessive SGB | P-value |
| --- | --- | --- | --- |
| Number, n | 10 | 18 |  |
| Male, n (%) | 8 (80.0) | 6 (33.3) | 0.02 |
| Mean age (SD) (years) | 50.3 (15.1) | 62.1 (15.4) | 0.04 |
| Mean BMI (SD) (kg/m²) | 25.5 (4.2) | 24.3 (4.9) | 0.56 |
| History of alcohol consumption, n (%) | 1 (10.0) | 1 (5.5) | 0.60 |
| History of smoking, n (%) | 3 (30.0) | 4 (22.2) | 0.49 |
| *H. pylori* infection n (%) | 0 (0) | 2 (11.1) | 0.41 |

BMI, body mass index; SGB, supragastric belching

Supplementary Table 2. Associations Between F-Scale Questionnaire (FSSG) Scores and Excessive SGB in Patients with True NERD

| FSSG Question No. | Excessive SGB | Non-excessive SGB | P-value |
| --- | --- | --- | --- |
| Reflux-related score, median (IQR) | 6.5 (6-13) | 13 (8.75-19) | 0.10 |
| Dyspepsia-related score, median (IQR) | 8 (6-9.5) | 8 (7.25-12.5) | 0.38 |
| Total score, median (IQR) | 14.5 (12.5-20.75) | 20.5 (16.25-31.5) | 0.14 |

FSSG; Frequency Scale for the Symptoms of GERD, GERD, gastroesophageal reflux disease; SGB, supragastric belching; IQR, interquartile range (25th–75th percentile)

|  | Excessive SGB | Non-excessive SGB | P-value |
| --- | --- | --- | --- |
| Number, n | 10 | 18 |  |
| Median (IQR) acid exposure time (%, daytime) | 10.15 (8.625-15.15) | 12.25 (7.425-20.075) | 0.76 |
| Median (IQR) acid exposure time (%, nighttime) | 4.8 (0.35-11.575) | 5.95 (0.025-8.575) | 0.88 |
| Median (IQR) acid exposure time (%, 24-hours) | 9.25 (6.55-14.625) | 8.5 (5.975-22.375) | 1.00 |
|  |  |  |  |
| Median (IQR) bolus exposure (%, daytime) | 5.45 (2.55-8.9) | 2.7 (1.875-7.65) | 0.43 |
| Median (IQR) bolus exposure (%, nighttime) | 0.25 (0.025-0.55) | 0.2 (0.025-1.55) | 0.87 |
| Median (IQR) bolus exposure (%, 24-hours) | 3.2 (1.75-7.875) | 2.55 (1.425-7.525) | 0.67 |
|  |  |  |  |
| Median (IQR) acid reflux, n (daytime) | 43.5 (31.5-74.75) | 28 (13.75-46.5) | 0.068 |
| Median (IQR) non-acid reflux, n (daytime) | 22 (11.25-47.5) | 12 (6.25-24.25) | 0.17 |
| Median (IQR) total reflux, n (daytime) | 82.5 (52-101.25) | 39.5 (29.25-59) | 0.027 |
|  |  |  |  |
| Median (IQR) acid reflux, n (nighttime) | 4.5 (0.25-6.5) | 2.5 (0.25-4) | 0.51 |
| Median (IQR) non-acid reflux, n (nighttime) | 1 (0.25-2.75) | 1.5 (0-2) | 0.98 |
| Median (IQR) total reflux, n (nighttime) | 7 (1-10.5) | 4 (2-11) | 0.94 |
|  |  |  |  |
| Median (IQR) total reflux, n (24-hours) | 82.5 (59.25-113.25) | 40.5 (31.5-87) | 0.046 |

Supplementary Table 3. Esophageal Acid and Bolus Exposure Times and Esophageal Reflux Episodes in Patients with True NERD

AET, acid exposure time; SGB, supragastric belching; IQR, interquartile range (25th–75th percentile)

Supplementary Table 4. Associations Between Excessive SGB and Esophageal Motility in Patients with True NERD

|  | Excessive SGB | Non-excessive SGB | P-value |
| --- | --- | --- | --- |
| Number, n | 10 | 18 |  |
| Median (IQR) LESP (mmHg) | 20.9 (16.2-28.075) | 11.8 (3.8-27.625) | 0.26 |
| Median (IQR) IRP (mmHg) | 7.5 (4.25-16.5) | 11.5 (7.25-21.75) | 0.14 |
| Median (IQR) DCI (mmHg-sec-cm) | 633.5 (318-1126.75) | 656.0 (408-1083.75) | 0.76 |
|  |  |  |  |

DCI, distal contractile integral; IRP, integrated relaxation pressure; LESP, lower esophageal sphincter pressure; SGB, supragastric belching; IQR, interquartile range (25th–75th percentile)
